# Healthy Firms: Constraints to Growth among Private Health Sector Facilities in Ghana and Kenya

**DOI:** 10.1371/journal.pone.0027885

**Published:** 2012-02-24

**Authors:** Nicholas E. Burger, Daniel Kopf, Connor P. Spreng, Joanne Yoong, Neeraj Sood

**Affiliations:** 1 RAND Corporation, Arlington, Virginia, United States of America; 2 Department of Economics, London School of Economics, London, United Kingdom; 3 The World Bank, Washington, D.C., United States of America; 4 School of Pharmacy, University of Southern California, Los Angeles, California, United States of America; Kenya Medical Research Institute - Wellcome Trust Research Programme, Kenya

## Abstract

**Background:**

Health outcomes in developing countries continue to lag the developed world, and many countries are not on target to meet the Millennium Development Goals. The private health sector provides much of the care in many developing countries (e.g., approximately 50 percent in Sub-Saharan Africa), but private providers are often poorly integrated into the health system. Efforts to improve health systems performance will need to include the private sector and increase its contributions to national health goals. However, the literature on constraints private health care providers face is limited.

**Methodology/Principal Findings:**

We analyze data from a survey of private health facilities in Kenya and Ghana to evaluate growth constraints facing private providers. A significant portion of facilities (Ghana: 62 percent; Kenya: 40 percent) report limited access to finance as the most significant barrier they face; only a small minority of facilities report using formal credit institutions to finance day to day operations (Ghana: 6 percent; Kenya: 11 percent). Other important barriers include corruption, crime, limited demand for goods and services, and poor public infrastructure. Most facilities have paper-based rather than electronic systems for patient records (Ghana: 30 percent; Kenya: 22 percent), accounting (Ghana: 45 percent; Kenya: 27 percent), and inventory control (Ghana: 41 percent; Kenya: 24 percent). A majority of clinics in both countries report undertaking activities to improve provider skills and to monitor the level and quality of care they provide. However, only a minority of pharmacies report undertaking such activities.

**Conclusions/Significance:**

The results suggest that improved access to finance and improving business processes especially among pharmacies would support improved contributions by private health facilities. These strategies might be complementary if providers are more able to take advantage of increased access to finance when they have the business processes in place for operating a successful business and health facility.

## Introduction

Most analysts interested in health care in Sub-Saharan Africa (SSA) are familiar with the fact that health care outcomes and health care access remain poor in SSA, and many SSA countries are struggling to meet the Millennium Development Goals (MDG) for health [1]. Some are also aware that both public and private providers—we define the “private sector” as including for-profit and not-for-profit providers—play an important role in providing health care to rich and poor populations alike [2]. Given these facts, it is unlikely that any pragmatic solution to increase health care access can be achieved without active participation of both the private and public health care sector. While much attention and resources have been devoted to the public sector, recently international donors and multinational organizations have also begun to focus their efforts on more effective support of the private sector. There are renewed efforts to work with the private sector and support improvement in the policy environment for health care providers (e.g., the World Bank Group *Health in Africa Initiative*).

However, private providers can only be part of a sustainable solution for improving access to good quality care if they have the ability to increase the quality and quantity of the services they provide; which is to say if they can operate and grow as a business. To design public policies that effectively improve the private health sector's contributions to national health systems, policymakers first need to understand the constraints facing the private sector. Increasing such contributions may, but need not, imply the growth of the private health sector in terms of its share of total health care provision, relative to the share of publicly provided care. And “growth” is to be understood here to refer primarily to the operations of individual facilities and their ability to expand. Understanding the capacity and limitations of the private sector is a crucial first step for evaluating whether the private sector can play a major role in meeting the growing health care needs of SSA and how to best support its ability to do so. However, as we discuss below, prior research on the challenges facing the private health sector and its capacity to grow is fairly limited.

This paper aims to fill this gap in the literature by providing new information about private health facilities and the barriers and obstacles they face as health *businesses*. We report results from the Health Provider Assessment Survey (HPAS), which gathered data on approximately 300 private and public facilities in Ghana and 300 similar facilities in Kenya in 2010. We evaluate the constraints facing private providers, including access to key infrastructure, personnel, and challenges dealing with the government. We also assess the capacity of private providers by examining their business processes and access to financial markets.

### Role of Private Sector

The appropriate role of the private sector in health care remains a much-debated and contentious issue. Critics of private sector participation argue that private providers offer poor quality of care [3]–[5]. However, poor quality of care is not unique to the private sector and might be endemic to health systems in less developed economies. For example, new evidence from a recent multi-country studies suggests that quality of care and provider competence is roughly equivalent in the public and private health sector [6]. Other critics are concerned about user fees charged by private health care providers, suggesting that such fees limit access to care among the poorest, consequently increasing disparities in health care utilization [7], [8]. The evidence here is also mixed. Some SSA countries charge for services in public facilities (for examples see: [9]), and there is no conclusive evidence that user fees in the public sector are lower than in the private sector [10].

In contrast, given that health systems are often resource-constrained, an alternative way to improve access to care is to acknowledge and build upon the opportunities and resources of an existing private health sector [11], [12], [2]. Recent work using data from 34 SSA countries finds that increased private sector participation is associated with improved access and reduced disparities in care between rich and poor as well as urban and rural populations [13]. The result persists after controlling for per capita GDP and maternal education, two confounding factors that could be correlated with increased private sector participation and improved health care access.

While the debate about appropriate role of the private health sector is unresolved, the need to address problems of poor health outcomes and access to care in SSA is urgent. Given the large role of the private health sector in most countries, a basic level of engagement by government is necessary [14]. Effective engagement with the private sector will need to address the constraints private health care providers face both as businesses and as health care providers. In other words, policies should aim to not only improve the quality of care in the private sector but also ensure that these providers can become and remain viable and self-sustaining while meeting the health care needs of the population. Nevertheless, policies that directly or indirectly lead to expansion of the private sector may have an ambiguous effect on equity and access (see, e.g., equity discussions in [14]). In this paper, we focus on issues related to health care providers as businesses and leave other issues for future research.

### Prior Research on Health Care Facilities

Over the past 30 years health facility surveys in the developing world have become a principal source of obtaining data on health service delivery, health expenditure, and quality of care. In this section we review the types of information collected through past surveys and where gaps exist. We also review research that has considered the business aspects of health facilities.

Most research on health service provision in SSA focuses on the public sector, and most previous health facility surveys have gathered data primarily from public facilities (e.g., the Public Expenditure Tracking Survey or the Nigeria Primary Health Facility Survey). Exceptions include the Quantitative Service Delivery Survey (QSDS) in Uganda, the Service Provision Assessment (SPA by MEASURE), and the facility component of the Indonesian Family Life Survey (IFLS), all of which include private health facilities. The SPA cover private facilities but do not focus on revenue and cost issues or the regulatory and business environment health facilities face. Other surveys have focused specifically on costs and efficiency. The QSDS assesses variation in cost-efficiency and resource use for public and private facilities in Uganda (2000) and Mali (2004). The IFLS health facility module, conducted periodically between 1993 and 2008, provides in-depth information on basic services and fees, and it includes vignette-style questions to assess health care quality. However, few surveys have looked at the characteristics of small health care providers, such as pharmacies or chemical sellers, which in many cases provide frontline care to patients seeking treatment (e.g., [15]). For more detailed information on health facility surveys see, for example, [16] on service delivery.

Private health facilities also need to remain viable and self-sustaining businesses. They do not produce the same types of goods and services as, for example, manufacturing or service sector firms, but they share many of the same challenges and constraints. Research on private health facilities in SSA has assessed business-related facility characteristics in the context of factors that affect consumers directly, such as user fees charged or the number of hours a facility is open each day. But less attention has been paid to basic business characteristics (e.g., access to capital) or the business environment (e.g., regulatory burden).

Researchers looking outside the health care sector, however, have developed advanced survey instruments, such as the World Bank's Enterprise Surveys (ES), to assess the multitude of factors that affect how firms make decisions. The ES cover dozens of countries and address a range of business and business-environment topics. They typically ask firms about their costs and revenues, experiences dealing with government officials, labor force capacity, and regulatory environment. However, these surveys focus only on manufacturing and small retail sectors, and the relevance of their results to health facilities is unknown. For example, the Ghana Manufacturing Enterprise Survey (multiple years) gathers detailed financial data from manufacturing firms about products sold, indirect costs, depreciation, loans and interest, capital investment, and labor costs. Non-health firm surveys provide insight into the types of data that are most relevant and most difficult to capture. In this paper we compare results for health care providers in Kenya and Ghana to surveys (i.e., ES) done for non-health firms in each country.

## Methods

The data used for the study come from the Health Provider Assessment Survey, which was administered in Ghana and Kenya during 2010. We surveyed a sample of health facilities in seven districts in Ghana and five districts in Kenya, with districts in each country purposively chosen to be geographically and economically diverse. We provide more detail on the sample below.

Because the survey focuses on both business and health topics, in many cases it was administered to different individuals within the facility, including medical staff and managerial staff. In some cases, one individual served as both the facility manager and principal medical staff. The modal respondent in Kenya is a pharmacist, while in Ghana the modal respondent was a business manager. These roles are not mutually exclusive, and “pharmacists” could also be the “business manager” and vice versa. Facilities in both countries had been operating for an average of approximately 16 years.

### HPAS sample characteristics

The data used for the study come from the Health Provider Assessment Survey, which was administered in Ghana and Kenya during 2010 by the study team. HPAS samples for each country were designed to capture a broad range of health facility types, focusing primarily on smaller, private sector firms.

In Ghana, the sampling frame was based on a 2010 census of health facilities in seven districts purposively chosen to be geographically and economically diverse, carried out by the Results for Development Institute. We excluded laboratories and medical device manufacturers and out of the remaining 647 facilities, we interviewed a random sample of 300 hospitals, clinics, nursing homes and pharmacies. Private hospitals and clinics were oversampled.

In Kenya, we constructed a census of health facilities in five districts also reflective of geographic and economic diversity, by combining a list of 1920 hospitals, clinics, and nursing homes compiled by the Ministry of Health and KEMRI-Wellcome Trust with a list of 1948 pharmacies from a retail census collected by TNS Opinion. Similarly, we interviewed a random sample of 300 hospitals, clinics, nursing homes and pharmacies drawn from this census, oversampling private hospitals and clinics.

Table 1 shows the final HPAS survey composition by provider type in each country. We note that response rates for the survey differed across countries—90 percent in Ghana and 69 percent in Kenya—but we do not have any evidence of differential self-selection affecting the final sample composition.

**Table 1:** HPAS sample composition by country.

	Kenya		Ghana	
	Public	Private	Public	Private
Hospital	1	10	8	21
Clinic	11	112	31	68
Pharmacy	1	145	0	92
Chemical Seller	0	6	0	80
Nursing/maternity home	0	7	0	0
Other	5	2	0	0
**Total**	**18**	**282**	**39**	**261**

In this study we focus on the subsample of private (for-profit and not-for-profit) facilities that provide clinical services and commodities, i.e., clinics and pharmacies. In both countries we have grouped prescribing drug sellers (“pharmacies”) and non-prescribing drug sellers (“chemical sellers”) into a single “pharmacies” category. In Kenya we classify nursing/maternity homes as clinics for the purpose of this paper. In Ghana, the analytical sample consists of 68 clinics and 172 pharmacies, and in Kenya 119 clinics and 151 pharmacies. Thus the analytical sample reflects private clinics and pharmacies surveyed in the seven districts in Ghana and five districts in Kenya.

We discuss briefly registration rates, which provides context for how to interpret the HPAS sample of firms. A basic activity required by most countries is registering a business with the appropriate authorities, and data on registration can provide some insight into the types of private sector facilities included in the HPAS. For private health facilities, government registration typically includes registering with the relevant health authority (e.g., healthy ministry) and the tax office. Table 2 shows registration rates in Ghana and Kenya for both health ministry and tax office registration. Registration rates are high in both countries—especially for clinics—but pharmacies in Kenya register at lower rates for both types of registration than clinics in Kenya and pharmacies in Ghana. We present this data to acknowledge that our health facility sample frame and analytical sample likely underrepresents informal (i.e., unregistered) facilities, and results should be interpreted as such.

**Table 2:** Registration rates by country and facility type.

	Kenya				Ghana			
	Clinics	N	Pharmacies	N	Clinics	N	Pharmacies	N
*Registered with health ministry*	95.0%	119	79.5%	151	98.5%	67	97.7%	172
*Registered with tax office*	89.9%	119	72.8%	151	95.5%	67	98.8%	171
*Facility size* *(avg. # rooms)*	5.7	119	2.0	150	8.7	68	1.7	172
*Employees* *(avg. #)*	7.1	119	3.5	151	12.7	68	3.8	172
*Has refrigeration equipment*	71%	119	55%	150	94%	68	51%	172
*Has sterilization equipment*	85%	119	39%	150	84%	68	3%	172

Finally, in the last two lines of Table 2 we report basic facility characteristics on building size, employment, and two measures of facility equipment to provide additional context for our sample. Clinics in Ghana have approximately nine rooms, compared to an average of six rooms for Keynan clinics. Pharmacies in both countries have approximately two rooms. Summary statistics for employees mirror the results for building size: Ghanaian clinics employ more staff (12.7) than Kenyan clinics (7.1), but pharmacies in both countries have relatively similar numbers of employees (3.5 and 3.8 for Kenya and Ghana, respectively). Finally, more than 70 percent of clinics have refrigeration equipment or sterilization equipment, although fewer clinics in Kenya have refrigeration equipment than their counterparts in Ghana. Almost no pharmacies in Ghana have sterilization equipment, compared to 38 percent of pharmacies in Kenya. Approximately 50 percent of pharmacies in both countries have refrigeration equipment.

### Survey Questions

The survey questions are grouped into five core sections: basic facility characteristics, barriers and obstacles to operating a business, the policy environment, financial information, and business process management. (Full versions of the questionnaires are available upon request.) In Ghana we included a supplemental section regarding the national health insurance scheme, and for Kenya there was a supplemental section specific to pharmacies. A final section asks enumerators to provide a basic assessment of the facility, including information on amenities and cleanliness.

#### Barriers and Obstacles to Operating the Facility

The HPAS asks providers to identify the element of the business environment that presents the biggest obstacle faced by the facility. Additional questions ask the provider about their experience with registration (both health and tax authorities), the time they spend dealing with government regulations, and their experiences with informal payments.

#### Financing and financial management

Providers were asked about the financial instruments they use to operate and expand their facilities. Specifically, the HPAS asks detailed questions on the process of applying for loans, including (a) whether facilities sought a loan from financial institution, (b) whether the loan was approved, and (c) if they did not seek a loan why not. Another question asks how facilities finance their day-to-day operations. Providers were also asked whether they had expanded their facility in the past three years and, if so, how they financed the expansion. Finally, there are a set of questions about sources of finance for day-to-day operations and the types of financial management tools the providers use (e.g., bank accounts, paper- or electronic-based accounting systems).

#### Human Resource and Quality Assurance Systems

The HPAS asks providers about the methods and systems they use to monitor and improve human resources and quality of care. Facilities report whether they use paper- or electronic-based medical records and patient management systems. Providers are also asked whether they routinely carry out practices to improve quality, including (a) continuing education, (b) disseminating clinical practices, (c) producing internal reports on care, and (d) preparing statistics on patient receipt of services.

## Results

In this section we summarize the data from the HPAS on firms' barriers to and capacity for growth. We begin by highlighting the self-reported obstacles that private health care providers believe most inhibit their ability to effectively operate. We then focus in more detail on business processes, revenue and expenses, and access to financial markets. We also include results that highlight other business environment challenges health facilities face in Ghana and Kenya.

### Barriers and obstacles to operation

**Figure 1:**
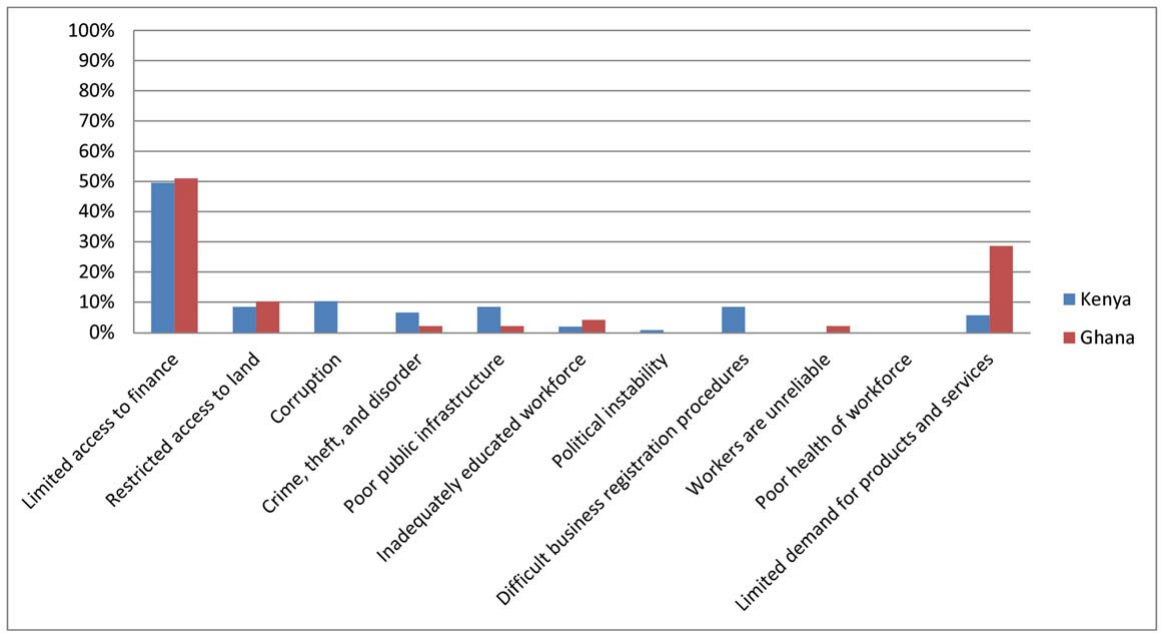
Note: Graph shows percent of firms responding that an obstacle is the most significant barrier that firm faces. Graph represents 107 clinics in Kenya and 49 clinics in Ghana. Source: Author calculations using HPAS data for Ghana (2010) and Kenya (2010).

**Figure 2:**
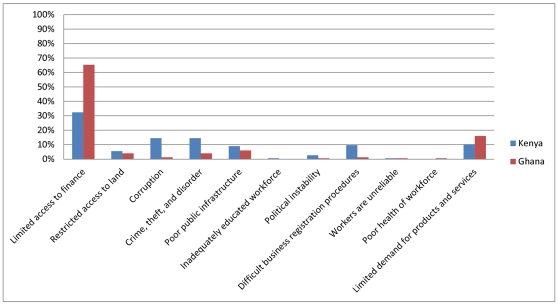
Note: Graph shows percent of firms responding that an obstacle is the most significant barrier that firm faces. Graph represents 145 pharmacies in Kenya and 150 pharmacies in Ghana. Source: Author calculations using HPAS data for Ghana (2010) and Kenya (2010).

The HPAS mirrors other, recent enterprise surveys in that it asks firms to identify the element of the business environment that poses the most significant barrier or obstacle to operating their facility. This question was adapted from the standard World Bank Enterprise Survey instrument, and it most closely mirrors the small or informal firm questionnaire. The results are reported in Figure 1 (clinics) and Figure 2 (pharmacies). A significant portion of clinics in Ghana (51 percent) and Kenya (49 percent) report that *limited access to finance* is the most significant barrier they face. Similarly, more pharmacies in both countries report *limited access to finance* is the most significant barrier, although the share in Ghana (65 percent) is substantially higher than in Kenya (32 percent). In Ghana, a relatively large share of facilities cites *limited demand for products and services* as the largest obstacle (clinics = 29 percent; pharmacies = 16 percent). In Kenya, similar shares of facilities (both clinics and pharmacies) report that *corruption*; *crime, theft, and disorder; poor public infrastructure*; and *difficult business registration procedures* are their largest business environment concerns. In Ghana, however, few other barriers rise to the top of firms' list of concerns, and in neither country are labor concerns the main obstacle for more than a handful of facilities. Below we discuss in more detail some of the major business environment barriers that firms identified.

**Figure 3:**
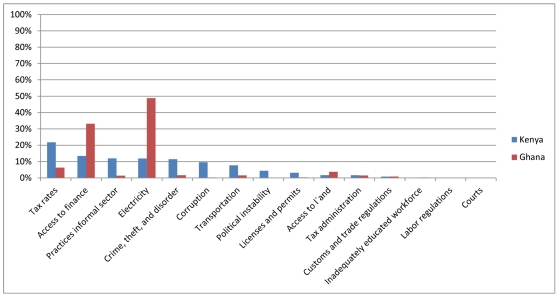
Note: Graph shows percent of firms responding that an obstacle is the most significant barrier that firm faces. Source: World Bank Enterprise Surveys for Kenya (2007) and Ghana (2007). Available online at: http://www.enterprisesurveys.org/. Data are for manufacturing firms.

The HPAS question on barriers and obstacles facing health facilities was modeled on the World Bank's Enterprise Surveys, which asks a similar question of small manufacturing firms. Although the two questions are not identical, it is possible to compare responses from manufacturing firms and health care providers in each country. Figure 3 presents the 2007 ES results for firms in Ghana (N = 494) and Kenya (N = 657). The most significant barriers and obstacles that manufacturing firms face are electricity in Ghana and tax rates in Kenya. These categories were not assessed in the HPAS, so a direct comparison with health care providers is not possible. Nevertheless, responses in categories that are assessed in both surveys reveal consistent patterns. Both health and non-health firms in Ghana report that access to finance is a significant barrier to operating their businesses. Similarly, *crime, theft, and disorder* ranks in the top five obstacles for both manufacturing firms and health care providers in Kenya. Notably, categories like *poor worker education* and *access to land* rank near the bottom of the list of barriers for both types of firms in both countries.

### Access to financial markets

As noted above, a plurality of private health care providers in Ghana and Kenya find access to finance a major factor limiting successful operation of their facility. Here we consider this issue in more detail, focusing on financing and access to capital questions in the HPAS. We first examine how facilities financed their day-to-day operations in the past year. This captures the sources of working capital private providers rely on to cover basic expenses. As Table 3 shows, most facilities, whether clinics or pharmacies, rely primarily on internal funds to fund daily activities. The results also show that only a small minority of health care providers use formal lending operations such as banks and microfinance for financing working capital needs.

**Table 3:** Sources of finance for day-to-day business operations.

	Kenya				Ghana			
	Clinics (%)	N	Pharmacies (%)	N	Clinics (%)	N	Pharmacies (%)	N
Internal funds	78	118	87	150	100	66	94	170
Credit from suppliers	19	118	20	150	48	64	67	168
Moneylender (informal)	6	118	12	149	0	64	1	166
Microfinance	14	118	6	150	0	64	6	166
Bank	14	118	09	150	8	64	5	166
Friends/relatives	19	118	29	150	3	64	7	166

*Notes: Responses refer to activity in the past year. Columns do not add to 100 as facilities were allowed to choose multiple sources of day-to-day financing.*

However, there is some variation across countries and facility types. Ghanaian providers of all types report less use of formal lending operations (i.e., microfinance and banks) than Kenya facilities; notably no clinics in Ghana reported financing day-to-day operations using microfinance. Similarly, Kenya facilities, especially pharmacies, report higher reliance on friends and relatives for short-term support. Perhaps the most notable feature about Ghanaian providers is the high rate of reliance on credit from suppliers for both clinics (48 percent) and pharmacies (67 percent). In contrast, only 19 percent of facilities in Kenya report using supplier-provided credit to fund daily operations, suggesting the supplier-facility financing relationship differs dramatically between the two countries.

Next we consider in more detail the process of applying for and acquiring loans from any type of formal financial institution in the past three years. We report results in Table 4. Less than a third of all facilities in either country reported applying for a loan, although application rates in Ghana were three times higher for clinics and two times higher for pharmacies than in Kenya. This contrasts with financing from lending institutions for day-to-day operations, where Kenyan facilities reported higher usage.

**Table 4:** Loan application and expansion activity in the past three years.

	Kenya				Ghana			
	Clinics	N	Pharmacies	N	Clinics	N	Pharmacies	N
Applied for a loan	11%	117	10	149	31	61	23	158
If applied, loan applications submitted (#)	2.6	12	2.0	15	2.5	17	2.1	36
If applied, applications rejected (#)	1	12	0.5	14	0.3	17	0.6	35
Facility had major expansion	29%	118	14%	151	28%	68	20%	171
Applied for a loan if had an expansion	9%	33	24%	21	53%	19	39%	31

*Notes: All questions ask about activity in the past three years.*

For those providers that applied for a loan in the past three years, clinics and pharmacies in both countries submitted roughly the same number of applications (between 2.0 and 2.5 per facility). On average, pharmacies in Ghana and Kenya saw one in four loan applications rejected. Rejection rates for clinics in Kenya were around 40 percent. In contrast, Ghanaian clinics had relatively few loans rejected (12 percent). Thus, Kenyan providers are less likely to apply for loans, but pharmacies that apply have broadly similar success rates to Ghanaian pharmacies, while clinics in Kenya are less successful.

One of the reasons a facility might apply for a loan is to fund a major purchase or facility expansion. The last two rows of Table 4 provide information about whether facilities had a “major expansion” (including expensive equipment purchase) in the past three years. Expansion rates are roughly the same across countries. When we look at loan application behavior for facilities that underwent a major expansion, we see that over half of clinics and nearly 40 percent of pharmacies in Ghana applied for a loan. Note that the data do not allow us to identify whether facilities applied for a loan to fund a major expansion. In contrast, only nine percent of clinics and 24 percent of pharmacies in Kenya who completed a major expansion applied for a loan from a financial institution. This suggests that firms making major capital investments in Kenya tend to finance their activities without loans.

The HPAS asked facilities who did not apply for loans to provide one or more reasons why they did not apply, and we report these results in Table 5. While it is not possible to disentangle whether firm quality, credit market constraints, interest rates, or financing alternatives definitively explain loan application behavior, there are some important patterns in the data. Pharmacies in Kenya report *lack of need* as the most common reason they did not apply for a loan (75 percent of facilities). For clinics in Kenya, the story is more complex. Clinics in both countries report similar “need” rates, but clinics in Kenya cite application complexity and collateral requirements more often than Ghanaian facilities as reasons they did not apply for a loan. Six percent of Kenyan clinics reported that they expected not to be approved due to registration status, while 11 percent reported the same expectation for other reasons. These data are consistent with both differential firm quality and differential market conditions across countries, but they counter the notion that Kenyan clinics are not interested in this type of financing.

**Table 5:** Reasons health facilities did not apply for a loan in the past three years.

	Kenya				Ghana			
	Clinics (%)	N	Pharmacies (%)	N	Clinics (%)	N	Pharmacies (%)	N
No need for a loan	53	105	75	135	51	41	38	118
Application procedures are complex	21	105	13	135	10	42	25	120
Interest rates are too high	33	105	24	135	33	42	47	121
Cannot meet collateral requirements	10	105	4	135	2	42	10	120
Expected to not be approved: not registered	6	105	2	135	0	42	1	121
Expected to not be approved: other reason	11	105	7	135	5	42	4	120
Other	4	105	1	134	12	42	6	120

*Notes: Responses refer to activity in the past three years. Columns do not sum to 100 as facilities were allowed to choose multiple reasons for not applying for a loan.*

### Government Effects on the Business Environment

Firms in Kenya reported that corruption was a major barrier to growth; here we assess the challenges firms face with corruption and other aspects of the business environment that involve dealing with the government. Table 6 summarizes facilities' experiences with corruption and red tape. As shown in Row 1, when asked what fraction of each 100 units of revenue a firm spent on informal payments to “get things done,” Kenyan clinics responded that 8 percent of revenue went to informal payments. Kenyan pharmacies were significantly lower, at 3.4 percent, but on average Kenyan health care providers spent more on informal payments than Ghanaian providers. These results are consistent with other, broad measures of corruption. For example, Ghana ranks 62^nd^ on Transparency International Corruption Perceptions Index 2010, while Kenya ranks 164 [17]. Note that 63 percent of all facilities in both countries report paying no informal payments to government officials.

**Table 6:** Facility experiences with corruption and red tape.

	Kenya				Ghana			
	Clinics	N	Pharmacies	N	Clinics	N	Pharmacies	N
Informal payments to govt officials (out of 100 revenue units)	8.0	114	3.4	148	0.1	58	0.5	124
Time spent dealing with govt regulations (out of 10 management hours)	3.5	114	3.1	146	1.1	56	1.0	143

*Notes: Percent of revenue spent on informal payments is out of every 100 local currency units of total revenue generated. Time spent dealing with government regulations is out of every 10 management hours. Time spent on government regulations response exclude those firms (6) that reported spending all 10 hours on government, as this is presumed to be infeasible. Neither top code appreciably affected the results. 66 facilities responded “don't know” (47) or “refuse” (19) to the informal payments question. 45 facilities responded “don't know” (42) or “refuse” (3) to the red tape question.*

A similar story emerges for our measure of “red tape.” A common question in enterprise surveys asks firms to report how much time they spend dealing with government officials (see e.g., [18]). Here, too, Kenyan health care providers report spending significantly more time dealing with government officials than Ghanaian providers, although this measure does not capture the quality or use of the time spent dealing with the government. A government with effective oversight procedures would appear to ‘burden’ health providers more than a government that had no oversight or inspective regime, but this would not imply that the health system in the latter was better. Recall that fewer Ghanaian firms reported that corruption was a major obstacle. The data on corruption experiences and time spent dealing with government officials are consistent with providers' relative assessments of obstacles to effectively operating their facilities in the two countries.

### Business processes and management tools

We also assess the extent to which facilities use common business tools to manage their patient records and financial accounts. Basic medical record systems are standard in developed countries. Table 7 reports use rates for both paper-based and electronic-based medical records system by country and facility type. Over 95 percent of clinics in Ghana and Kenya report using paper based patient record systems. The use of electronic patient records is relatively low, especially in Kenya where only 31 percent of clinics report using electronic patient records. And less than one in five pharmacies report using electronic patient records.

**Table 7:** Use rates for health and business management systems.

	Kenya				Ghana			
	Clinics (%)	N	Pharmacies (%)	N	Clinics (%)	N	Pharmacies (%)	N
Paper-based patient record system	95	119	79	151	96	68	46	166
Electronic-based patient record system	31	119	15	151	57	68	19	166
Paper-based accounting system	83	119	82	151	84	68	78	171
Electronic-based accounting systems	34	119	21	151	65	68	37	171
Paper-based inventory system for drugs and medical supplies	89	119	80	151	90	68	81	170
Electronic-based inventory system for drugs and medical supplies	29	119	19	151	54	68	36	170
CPA audit	36	119	38	151	66	65	38	172

*Notes: Percentages reflect fraction of facilities responding that they use each health or business management process.*

Table 7 reports how often providers use financial management systems, another basic tool in running a business. Once again, reported use rates for paper-based systems are relatively high, around 80 percent for all providers. In contrast, use of electronic systems is much lower, especially for pharmacies and clinics in Kenya. The next two rows of Table 7 report facility use of paper- and electronic-based inventory systems for drugs and medical supplies. Facilities were also asked whether they have hired a certified accountant to audit their facility's finances in the past year. As shown in the final row of Table 7, in Kenya, 36 percent clinics report hiring an accountant, while use rates are higher for clinics in Ghana (66 percent) and roughly the same for pharmacies (38 percent).

Finally, the HPAS asked facilities to report whether they used specific tools in the areas of human resource management and quality control. The goal is to ascertain whether facilities undertake activities designed to improve human capital or provide information—either for internal or external use—on basic provider behavior. These include sending staff for continuing medical education (CME), providing clinical guidelines to staff, and producing summary data on services provided to patients. As Table 8 shows, the rates at which facilities report using these tools vary by country and provider type. Notably, clinics report undertaking these activities at higher rates than pharmacies, but facilities of each type report similar usage across countries. The exception is for pharmacies in Ghana, which report producing summary data on patient care at lower rates than their counterparts in Kenya. It is also worth noting that it is difficult to benchmark these numbers; nevertheless, there is clear room for improvement in some areas. For example, CME rates are approximately 50 percent or lower, suggesting half or more facilities do not provide opportunities for staff to maintain or enhance their medical knowledge.

**Table 8:** Use of human resource and quality assurance systems.

	Kenya				Ghana			
	Clinics (%)	N	Pharmacies (%)	N	Clinics (%)	N	Pharmacies (%)	N
Send medical staff to continuing education	47	119	31	149	55	66	36	171
Disseminate clinical practice guidelines to staff	66	118	36	149	78	67	44	171
Produce internal report on care provided to patients	67	119	37	149	67	67	18	170
Prepare statistics on how many patients received key services	64	119	37	149	70	67	15	170

*Notes: Percentages reflect fraction of facilities reporting that they carry out each of the human resource and quality assurance activities.*

## Discussion

Historically, a main approach for increasing health care provision in the developing world has been to increase public provision. But efforts to date have not enabled many SSA countries to meet key health outcome targets, such as the MDGs. There is interest from policy makers and donors in using the private health sector to improve health outcomes, but research on the state of private health care providers and the constraints they face is limited. We present data from a health facility survey, administered to private providers in Ghana and Kenya, that describes the business aspects of private health care providers.

The data suggest that access to capital is the largest impediment facing private providers. Few providers use formal institutions for financing working capital. More detailed analysis suggests that firms in Kenya and Ghana have very different experiences when it comes to obtaining financing and loans, with Kenyan providers applying for loans at lower rates than their counterparts in Ghana. Although many facilities report not needing formal loans, a substantial fraction of providers in both countries cite lack of information as an impediment to applying for financing. Government policies could help reduce information barriers and allow firms to better assess the benefits of financing and their ability to obtain it. However, concurrent research using the same survey data suggests that this is not currently happening: only four percent of private providers in Kenya report receiving any form of technical assistance from the government regarding loan application processes—in Ghana no firms report receiving such assistance [19].

Corruption and red tape is also a significant barrier for day to day operations of private health care providers. Kenyan facilities report higher costs associated with corruption and spend more time dealing with government regulations than Ghanaian facilities. In Ghana, corruption costs are lower, a result consistent with other measures of the relative corruption levels in each country. We cannot assess the impact of corruption on health provision by private facilities, but the data suggest this is an area that may warrant attention by the Kenyan government. The data also suggest “red tape” may be a problem in Kenya, although it is important to acknowledge that reducing the amount of time providers spend dealing with government regulation is not unambiguously desirable. In Ghana the government could focus on improving the business environment by relaxing other constraints, such as through better public infrastructure.

Finally, the data indicate that health care providers could potentially benefit from adopting better business processes. This is especially true for pharmacies. Few pharmacies use electronic patient records, electronic accounting systems, or electronic records for inventory control. These providers also report relatively weak human resource and quality assurance systems. In contrast, clinics in Kenya and Ghana report high usage rates of key business processes, including accounting and patient records systems. Similarly, clinics report relatively high usage rates of tools to improve quality of care.

Overall the results suggest that improved access to finance and improving provider business processes might be complementary strategies. In other words, providers will be more able to take advantage of increased capital flows if they have the processes and tools in place for operating a successful business and health care facility.
